# C–H Bond Activation and C–C Coupling of Methane on a Single Cationic Platinum Center: A Spectroscopic and Theoretical Study

**DOI:** 10.1021/acs.inorgchem.2c01328

**Published:** 2022-07-12

**Authors:** Frank J. Wensink, Noa Roos, Joost M. Bakker, P. B. Armentrout

**Affiliations:** † Institute for Molecules and Materials, FELIX Laboratory, Radboud University, Toernooiveld 7, Nijmegen 6525 ED, The Netherlands; ‡ Department of Chemistry, University of Utah 315 South 1400 East, Room 2020, Salt Lake City, Utah 84112, United States

## Abstract

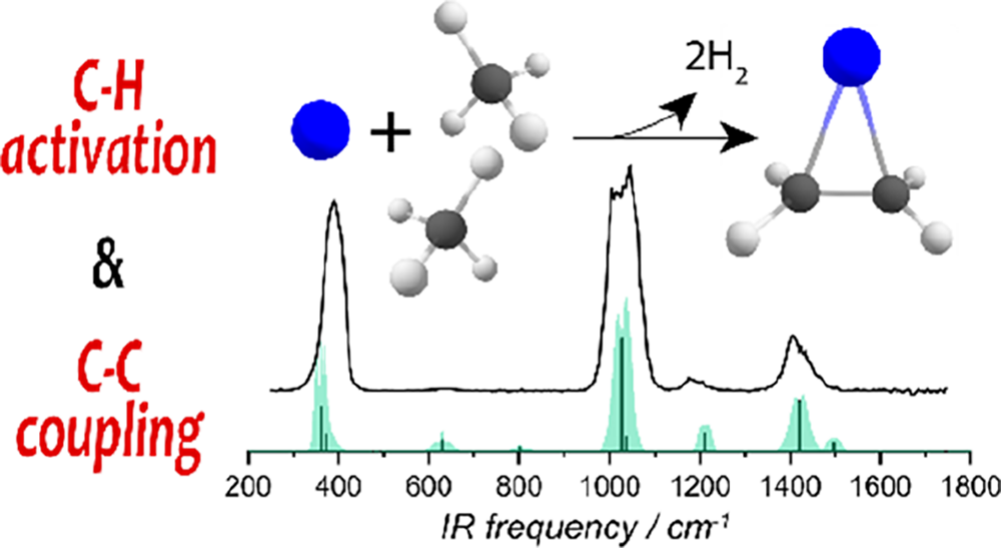

We spectroscopically investigated the activation products resulting from reacting one and multiple methane molecules with Pt^+^ ions. Pt^+^ ions were formed by laser ablation of a metal target and were cooled to the electronic ground state in a supersonic expansion. The ions were then transferred to a room temperature ion trap, where they were reacted with methane at various partial pressures in an argon buffer gas. Product masses observed were [Pt,C,2H]^+^, [Pt,2C,4H]^+^, [Pt,4C,8H]^+^, and [Pt,2C,O,6H]^+^, which were mass-isolated and characterized using infrared multiple-photon dissociation (IRMPD) spectroscopy employing the free electron laser for intra-cavity experiments (FELICE). The spectra for [Pt,2C,4H]^+^ and [Pt,4C,8H]^+^ have several well-defined bands and, when compared to density functional theory-calculated spectra for several possible product structures, lead to unambiguous assignments to species with ethene ligands, proving Pt^+^-mediated C–C coupling involving up to four methane molecules. These findings contrast with earlier experiments where Pt^+^ ions were reacted in a flow-tube type reaction channel at significantly higher pressures of helium buffer gas, resulting in the formation of a Pt(CH_3_)_2_^+^ product. Our DFT calculations show a reaction barrier of +0.16 eV relative to the PtCH_2_^+^ + CH_4_ reactants that are required for C–C coupling. The different outcomes in the two experiments suggest that the higher pressure in the earlier work could kinetically trap the dimethyl product, whereas the lower pressure and longer residence times in the ion trap permit the reaction to proceed, resulting in ethene formation and dihydrogen elimination.

## Abstract 2

Methane is reacted with gas-phase atomic platinum cations in a room temperature ion trap, and the resulting product ions are characterized using IR light of the Free Electron Laser for Intra-Cavity Experiments (FELICE). We observed the formation of PtCH_2_^+^, Pt(ethene)^+^, and Pt(ethene)_2_^+^. These findings demonstrate the C−H bond activation and C−C coupling of methane molecules.

## Introduction

Large amounts of methane are present in natural gas, but its great stability hinders widespread utilization by the chemical industry. Methane must be activated before it can be converted to a higher-value chemical such as methanol or ethene. Currently, methane is first thermally cracked, forming synthesis gas, before it can be converted into, for example, synthetic fuels via the Fischer–Tropsch process employing Fe- or Co-based catalysts.^1^ These processes require high temperatures and pressures, which cause the overall process to be energy inefficient. Indeed, Crabtree has pointed out, even though organometallic CH bond activation dates to 1962, there remains no “general, selective, efficient catalytic functionalization reactions of unactivated sp^3^ CH bonds”.^2^ In the gas phase, studies of alkane activation by metal cations date to the seminal work of Ridge in 1979, who observed the facile C–H (and C–C) bond activation of butanes by Fe^+^.^3^

In recent years, there have been many investigations of how transition metals (TMs) interact with methane.^4−6^ Studying these systems under well-defined conditions with highly sensitive mass-spectrometric methods can offer detailed information on reaction mechanisms and the electronic structure needed on the metal center that allows facile C–H bond activation. In such studies, it was found that several third-row TM cations are capable of dehydrogenating methane at room temperature.^7−18^ The structures of several [TM,C,2H]^+^ products were recently identified using infrared multiple-photon dissociation (IRMPD) action spectroscopy. Comparisons to theory identified TM^+^–CH_2_ carbene structures for Ta^+^, W^+^ (both with agostic distortions), and Pt^+^ (having the classic C_2v_ structure), and H–TM^+^–CH hydrido carbyne structures for Os^+^ and Ir^+^, where reaction with the latter metal ion also yielded a minority carbene product.^16,19−21^ In 1990, Irikura and Beauchamp observed that several of these third-row TM cations could also dehydrogenate multiple methane molecules, presumably by oligomerization.^7,22,23^ For Pt^+^, they reported dehydrogenation of up to five methane molecules but only characterized the first step, i.e., Pt^+^ + CH_4_ → PtCH_2_^+^ + H_2_ as facile. The structures of the products resulting from multiple methane dehydrogenations by any metal cation were never determined directly. Wheeler et al. studied the reaction products of Pt^+^ and Ir^+^ with multiple methane molecules formed in a helium buffer gas under relatively high helium pressure conditions using IRMPD spectroscopy.^24,25^ For the reaction with Pt^+^, they identified the [Pt,3C,10H]^+^ and [Pt,4C,14H]^+^ products formed as Pt(CH_3_)_2_(CH_4_)^+^ and Pt(CH_3_)_2_(CH_4_)_2_^+^, where the spectroscopy was enabled by the IR-induced loss of methane molecules.^24^ On the basis of quantum chemical calculations, they also concluded that Pt(CH_3_)_2_^+^ was not the global minimum structure for [Pt,2C,6H]^+^, with species such as (H_2_)Pt(C_2_H_4_)^+^ being significantly more stable. Therefore, the reaction of PtCH_2_^+^ with methane could form both Pt(CH_3_)_2_^+^ and (H_2_)Pt(C_2_H_4_)^+^ exothermically, but the barrier leading to the first species is below the energy of the PtCH_2_^+^ + CH_4_ reactants, whereas that for the second product is above it, preventing its formation. In an earlier theoretical study, Diefenbach et al. already concluded that PtCH_2_^+^ should react with methane to form a Pt(C_2_H_4_)^+^ product rather than the bis-carbene Pt(CH_2_)_2_^+^.^26^

In the current study, we reinvestigate the potential of gas-phase Pt^+^ ions to activate multiple methane molecules. In contrast to previous work, the reactions take place in a radio-frequency (RF) ion trap, allowing more control over the methane partial pressure and reaction times and, consequently, over the product distribution formed. The lower pressure reaction conditions in our RF ion trap lead to the formation of other product species than those observed by Wheeler et al. in the molecular beam environment.^24^ In our experiment, the reaction conditions are a closer match to the single-collision conditions of the experiments by Irikura and Beauchamp.^7^ After a fixed reaction time in our RF ion trap, all ions were transferred to the Fourier-transform ion cyclotron resonance mass spectrometer (FTICR-MS) coupled to the Free Electron Laser for IntraCavity experiments (FELICE).^27−29^ Here, the high photon flux provided by FELICE can be employed to fragment the [Pt,2C,4H]^+^ and [Pt,4C,8H]^+^ ions formed, something Wheeler et al. could not achieve with the conventional FELIX beam line.^24^ Finally, use of the FTICR-MS instrument allows products of interest to be mass-isolated, foregoing the need to rely on messenger tagging and enhancing the experimental sensitivity. Comparison of the experimental IRMPD spectra with theoretical calculations then permits the unambiguous identification of [Pt,2C,4H]^+^ and [Pt,4C,8H]^+^ as species containing one and two ethene ligands, thereby documenting the dehydrogenation and C–C coupling of multiple methane molecules on the single platinum center.

## Methods

### Experimental Section

The experiments were carried out in an FTICR-MS instrument coupled to a FELICE beamline^27,28^ with a laser vaporization source described earlier.^29,30^ Pt^+^ ions were produced by irradiating a rotating Pt disk by a frequency-doubled Nd:YAG laser operating at 30 Hz and producing ∼3 mJ pulses. To cool the ions formed and to entrain them in a supersonic expansion when exiting the source region, a pulse of He carrier gas was injected through a piezoelectric valve prior to ablation. Ions were then transferred by ion optics and a quadrupole mass filter operated in guiding mode to a sectioned RF linear quadrupole ion trap with rectangular electrodes and two DC-biased circular end electrodes with a hole to admit or expel ions. The ion trap is enclosed by a room temperature cylinder that separates it from the background vacuum. Ions were thermalized by collisions with argon, introduced through a leak valve into the cylinder at a pressure of approximately 8 × 10^–4^ mbar measured with a Baratron pressure gauge directly connected to the cylinder. Methane was let in via a second leak valve. A methane partial pressure ranging from 2 × 10^–5^ up to 8 × 10^–4^ mbar was used to maximize the intensity of the ionic species of interest.

After reacting over a period of approximately 200 ms, all ions formed were expelled from the trap by reducing the voltage on one of the end electrodes. Ions were subsequently deflected by 90° using a DC quadrupole bender and transferred to one of four ICR trapping cells coinciding with the center of the magnetic field produced by a 7 T superconducting magnet. Here, unwanted masses were ejected via a combination of single frequency, chirped, and stored-waveform inverse Fourier transform (SWIFT) pulses.^31^ After mass isolation, the ions of interest were irradiated by a single macropulse of the FELICE free-electron laser.^27^ Upon resonant vibrational irradiation, photofragmentation was induced, after which all ions present were detected. The molecular response to the IR laser is represented by the fragmentation yield *Y*_F_, defined as
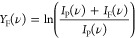
where *I*_P_(ν) and *I*_F_(ν) represent the mass spectral intensities of precursor and fragment species as a function of IR frequency ν. Experimental spectra were acquired by plotting the fragmentation yield as a function of ν. IR spectra were normalized on the macropulse energy inferred from outcoupling a fraction of the intracavity light, which was also used for wavelength calibration. FELICE macropulses had a pulse energy of approximately 0.3 J when investigating [Pt,2C,4H]^+^ over the whole spectral range and 0.7 J for [Pt,4C,8H]^+^. Spectral bandwidths range from 0.9% of the central frequency in the low-frequency region to 0.33% in the high-frequency region. ICR cell 1 coincides with the focus of FELICE and thus has the highest photon fluence. ICR cells 2, 3, and 4 are 100, 200, and 300 mm away from the focus of FELICE, respectively. The FELICE Rayleigh length of 82 mm leads to an IR fluence in cell 4 that is 14 times lower than in cell 1.^27,29^

### Computational Section

To interpret the experimental IR spectra and to rationalize product formation pathways, density functional theory (DFT) calculations were carried out. Calculations were performed using the Gaussian16 software package^32^ at the UB3LYP level of theory^33,34^ using the def2-TZVPPD basis set, which had previously been proven accurate for describing the systems of interest.^24^ To benchmark our methods, we calculated the binding energies of Pt^+^–H, Pt^+^–CH_2_, and Pt^+^–CH_3_ to be 2.95, 5.04, and 2.85 eV, respectively, compared to experimentally determined values of 2.81 ± 0.05, 4.80 ± 0.03, and 2.67 ± 0.08 eV, respectively.^17,35^ For platinum, this basis set uses an effective core of [Kr]4d^10^4f^14^ and explicitly treats the 5s, 5p, 6s, and 5d valence electrons. Both doublet and quartet spin states were investigated. For all structures, harmonic frequencies were calculated to ascertain that they are either a true minimum or a first-order transition state. Intrinsic reaction coordinate calculations were performed to make sure that the transition states connect the desired intermediates. All frequencies used were scaled by a factor of 0.97 to account for anharmonicity and the redshift associated with the IRMPD process. This value is in between 0.939 and 0.983, as found earlier for similar systems.^16,24^ The calculated spectra were convoluted with a Gaussian line shape function with a FWHM of 20 cm^–1^ for comparison to experimental spectra. To assess potential broadening resulting from the rotational substructure of vibrational transitions for selected species, we simulated rotational profiles for each vibrational band by assuming pure *a*-, *b*-, or *c*-type transitions and identical rotational constants (retrieved from the DFT calculations) for ground and excited vibrational states. To do so, we used Prof. L. Meerts’ homebuilt software for diagonalizing the rotational Hamiltonian and convoluted the individual transitions, weighted by a single Boltzmann temperature factor, with a 0.9% FWHM Gaussian line shape function. Finally, all calculated energies reported in this work were zero-point energy-corrected using unscaled vibrational frequencies.

## Results and Discussion

### Products Formed in the Reaction of Pt^+^ with Methane

**Figure 1:**
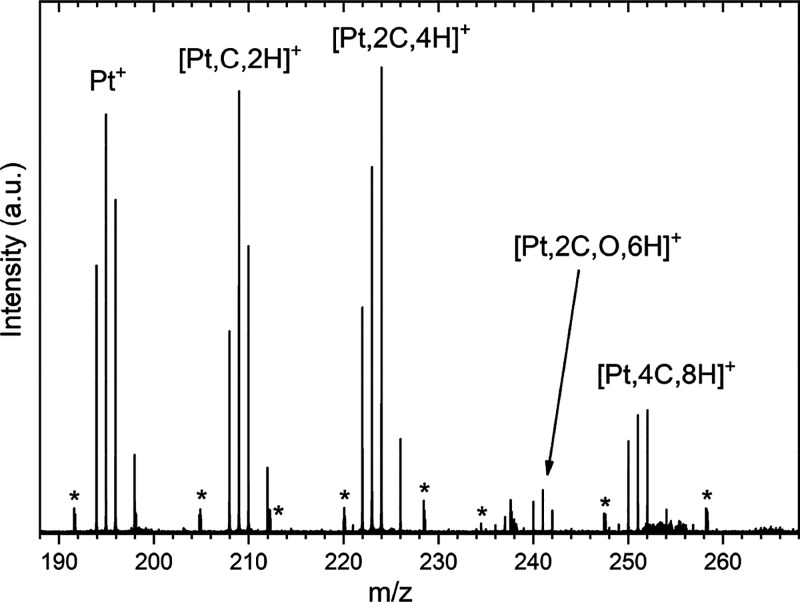
Composite mass spectrum of species formed by reacting Pt^+^ with CH_4_ at various partial pressures of CH_4_ in the ion trap. Artifacts resulting from electrical noise pickup are denoted by an asterisk (*).

Figure 1 shows a composite mass spectrum of ions observed when reacting Pt^+^ ions with methane in the ion trap, created by adding several mass spectra with varying methane partial pressures in the ion trap together. Pt^+^ ions dominate in absence of methane, showing its characteristic isotopic distribution of masses 194, 195, 196, and 198 amu (33, 34, 25, and 7% natural abundance, respectively). As the methane partial pressure is increased, [Pt,*n*C,2*n*H]^+^ (*n* = 1, 2, 4) product ions gradually appear, with [Pt,4C,8H]^+^ being evident when both argon and methane have a partial pressure of approximately 8 × 10^–4^ mbar in the ion trap. Interestingly, no clear sign of a [Pt,3C,6H]^+^ product was observed. Instead, a set of minor products at *m/z* = 240, 241, and 242 was observed, 46 Da higher in mass compared to the three primary Pt isotopes. This product could be identified mass-spectrometrically as [Pt,2C,O,6H]^+^, and for reasons explained below, this appears to be a water adduct of the [Pt,2C,4H]^+^ product, presumably formed by a trace water contamination in either the argon or methane lines. When spectroscopically investigating a single ionic species using IR light, we ensured that all other species were ejected from the ICR cell. Only the ^194^Pt and ^195^Pt isotopes were used to obtain IRMPD spectra of the molecular ions examined in order to avoid ambiguities associated with products that had not dehydrogenated.

In stark contrast to the experiments carried out by Wheeler et al. in a molecular beam environment,^24^ no [Pt,*n*C,(2*n* + 2)H]^+^ (*n* = 1–4) products were observed. Our initial interpretation for this difference is that this is a result of the significantly longer reaction times (200 ms versus ∼100 μs) and the lower total pressures (10^–4^ to 10^–3^ mbar versus an estimated pressure exceeding 10 mbar). Such differences are in accordance with results reported for similar experimental designs to the one used by Wheeler et al.^36−38^ The reaction in the higher pressure flow tube may thus trap intermediate reaction products. A final difference between the two experiments is that in the expansion into vacuum to form a molecular beam, products may be complexed with extra methane molecules.

### IR Spectroscopy of [Pt,C,2H]^+^

**Figure 2:**
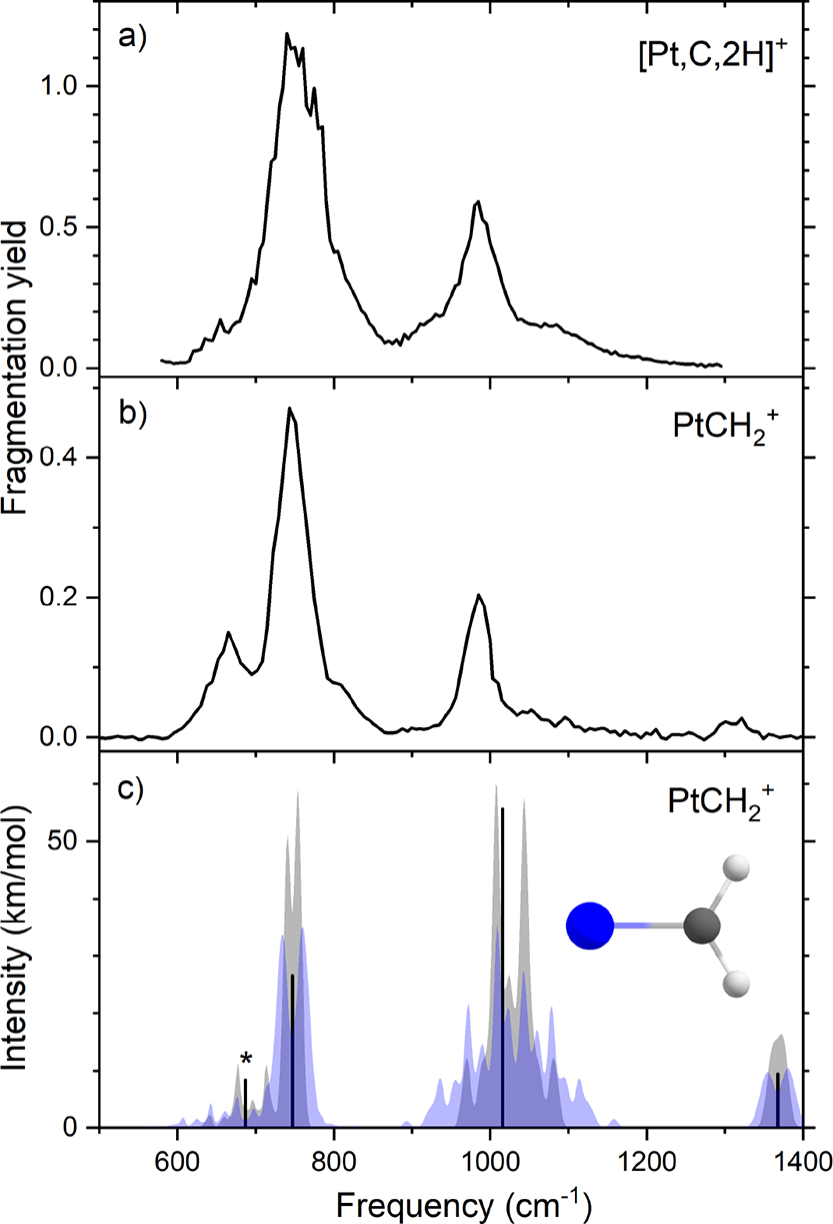
(a) Experimental IRMPD spectrum of [Pt,C,2H]^+^ formed in the ion trap at room temperature. (b) Experimental IRMPD spectrum of [Pt,C,2H]^+^ reported by Lapoutre et al.^16^ (c) Room temperature (blue) and 77 K (gray) simulated spectrum of the PtCH_2_^+^ (^2^A_1_) structure, including rovibrational band shapes and the harmonic frequencies in black. The asterisk (*) denotes a frequency that was shifted down 40 cm^–1^ before scaling by 0.97.

As a first test to check whether the products formed in the current experimental instrument are the same as those formed in the higher-pressure flow tube of Lapoutre et al.^16^ we compare the IRMPD spectrum for the [Pt,C,2H]^+^ product with the spectrum of PtCH_2_^+^ reported earlier. Figure 2a shows the IRMPD spectrum recorded in ICR cell 3 for [Pt,C,2H]^+^, with H_2_ loss as the fragmentation channel. It is compared to the IRMPD spectrum recorded using a molecular beam experiment as shown in Figure 2b.^16^ Both spectra are quite similar, dominated by an intense band at 749 cm^–1^ and a weaker band at 977 cm^–1^. Overall, the bands in the current spectrum, especially the band at 749 cm^–1^, appear a bit broader, and the peak at 977 cm^–1^ seems to have more pronounced shoulders on both sides. The band at 665 cm^–1^ observed in the earlier spectrum is not obvious in the present spectrum, although it could be buried in the tail of the 749 cm^–1^ band. The band at 749 cm^–1^ is assigned to two overlapping modes: the Pt–C stretch calculated at 747 cm^–1^ and the in-plane CH_2_ rocking mode calculated at 726 cm^–1^, with the latter having an intensity three times smaller. The 977 cm^–1^ experimental band corresponds to the out-of-plane CH_2_ wagging mode calculated at 1016 cm^–1^.

Rovibrational simulations at 77 K (gray) and room temperature (blue) are shown in Figure 2c. Here, it can be seen that the absence of the 665 cm^–1^ band, the appearance of a broader shoulder structure for the 977 cm^–1^ band, and the broadening of the 749 cm^–1^ band all can be rationalized by the higher temperature at which the current spectrum was recorded. Although both reactions have taken place at room temperature, the spectrum in Figure 2b was taken after the reactive mixture was expanded into a vacuum, thereby undergoing significant cooling of the internal degrees of freedom, whereas the ions formed in the current experiment were transferred without such cooling. We conclude that the room-temperature simulation for this experiment is accurate enough to explain most features. It must be noted that as in a previous publication, the *b*-type transition associated with the in-plane C–H wagging vibration (denoted with an asterisk in Figure 2c) was calculated at 748 cm^–1^ (unscaled) but has been shifted down by 40 cm^–1^ before scaling by 0.97 to better match the observed spectrum.^18^

### IR Spectroscopy of [Pt,2C,4H]^+^

**Figure 3:**
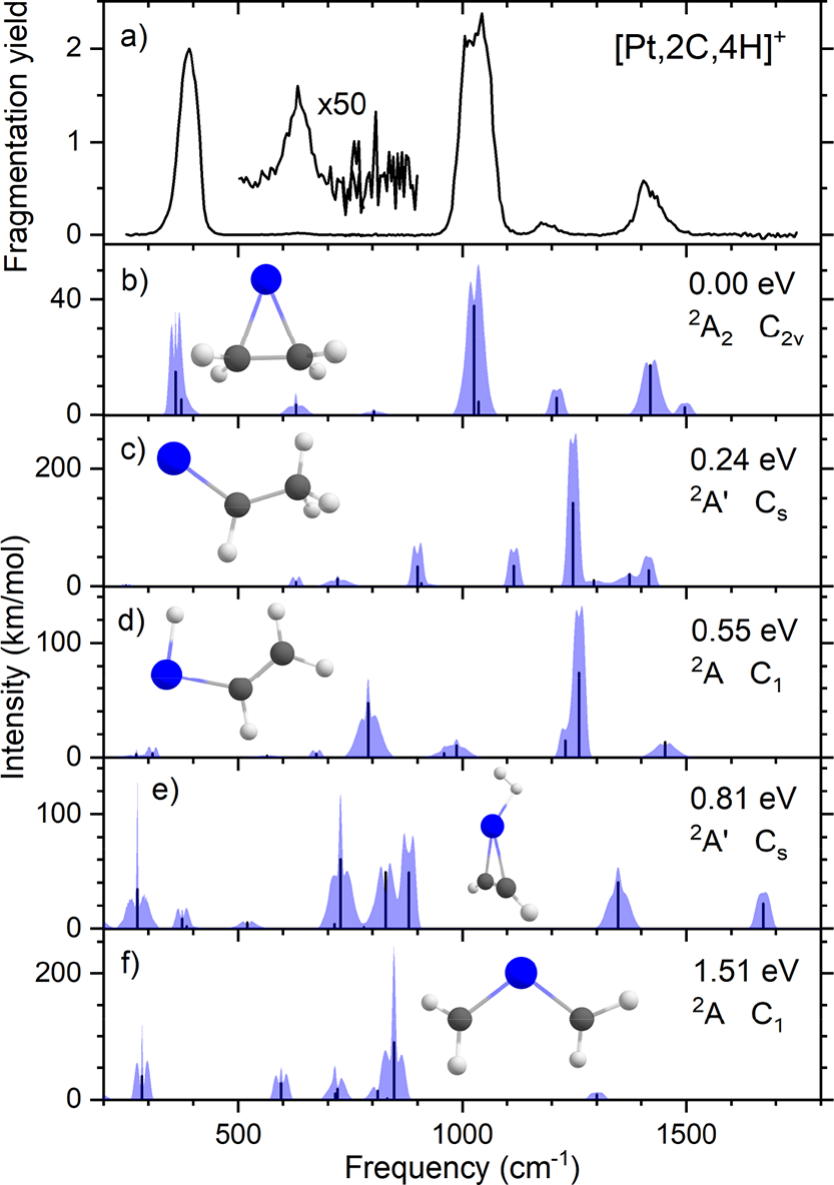
(a) Experimental IRMPD spectrum of [Pt,2C,4H]^+^. (b–f) Calculated spectra of different [Pt,2C,4H]^+^ isomers including rovibrational simulations at room temperature accompanied by molecular structures, relative energies, electronic ground states, and point groups.

Figure 3a shows the IRMPD spectrum of [Pt,2C,4H]^+^ (*m/z* = 222, 223). It was recorded using FTICR cell 3 between 250 and 780 cm^–1^ and using FTICR cell 4 between 735 and 1745 cm^–1^ via H_2_ loss into the *m/z* = 220, 221 mass channels. At higher IR intensities, we observe a second loss channel, namely, into the bare Pt^+^ mass channel as shown in Figure S1. Two strong IR bands are present at 391 and 1030 cm^–1^, and a medium-strong band is found at 1404 cm^–1^ with a tail toward the blue. Weaker bands are found at 634 and 1186 cm^–1^.

The experimental spectrum is compared to calculated spectra of several trial structures. We found seven structures for the [Pt,2C,4H]^+^ species, five of which are shown in Figure 3b–f, including simulated rovibrational envelopes. The other two, one being an isomer with very high energy and the other being a rotational isomer of the structure shown in Figure 3d, are shown in Figure S2. The lowest-energy structure is the PtC_2_H_4_^+^ complex (ethene ligand), a structure with C_2v_ symmetry where Pt lies 2.04 Å above the center of the C–C bond. It is located 0.53 eV below the PtCH_2_^+^ + CH_4_ reactants when the elimination of H_2_ is assumed. A PtCHCH_3_^+^ complex (ethylidene ligand) is found to be 0.24 eV higher in energy than the ethene complex. This structure can be seen as a carbene structure where one of the hydrogen atoms is replaced by a methyl group. Transfer of a hydrogen from the methyl group to the platinum results in a HPtC_2_H_3_^+^ species lying 0.55 eV above the PtC_2_H_4_^+^ structure and slightly above the energy of the reactants. A similar structure in which the H ligand lies *trans* to the vinyl ligand (instead of the *cis* orientation shown in Figure 3d) was found to lie 0.01 eV higher in energy and has a very similar spectrum (Figure S2). Transfer of another hydrogen atom to the platinum results in a (H_2_)PtC_2_H_2_^+^ structure 0.81 eV above the ethene complex. Finally, the complex with two carbene groups, Pt(CH_2_)_2_^+^, lies 1.51 eV above the PtC_2_H_4_^+^ complex. The lowest quartet species, PtC_2_H_4_^+^ (^4^A″), lies 2.15 eV above its doublet counterpart.

When we compare the calculated IR spectra of the different isomers with the experimentally obtained IRMPD spectrum of [Pt,2C,4H]^+^, by far, the best match is observed for PtC_2_H_4_^+^. It is the only structure offering predictions for all bands observed. Three of the bands predicted are readily attributed to the IR-active vibrations of ethene, which are found at 826, 949, and 1444 cm^–1^ in the free molecule.^39^ The most intense band in the experimental spectrum (1030 cm^–1^) can be assigned to the ethene-asymmetric CH_2_ wagging mode (in the Pt–C–C plane, 949 cm^–1^ in free ethene) predicted at 1027 cm^–1^ for the complex. Its symmetric counterpart, symmetry-forbidden in ethene (but Raman-active at 943 cm^–1^), carries IR intensity because of the presence of Pt and is predicted at 1037 cm^–1^ with an intensity eight times lower than the IR-active band. Both frequencies are slightly blueshifted upon binding to Pt^+^, as explained below. The asymmetric wagging mode is a *b*-type transition that, when its rovibrational envelope is simulated (see Figure 3b), even explains the double-peaked maximum observed. The experimental band at 1404 cm^–1^ is assigned to the concerted (out-of-phase) ethene CH_2_ scissoring mode (1444 cm^–1^ in free ethene) predicted at 1420 cm^–1^. This band has a shoulder on the high-energy side that can be assigned to the C–C stretch vibration predicted at 1497 cm^–1^. Because the C–C stretch vibration is found by Raman spectroscopy at 1623 cm^–1^ in free ethene, this mode is affected by the complexation as discussed further below.^39^ The out-of-phase in-plane CH_2_ rocking mode, weak in free ethene at 826 cm^–1^, does not gain any intensity and is predicted at 803 cm^–1^; from the inset of Figure 3a, it can be seen that no sign for this band is observed experimentally. However, when irradiating at higher intensities (Figure S1b), a very weak band is observed at this frequency.

The intense experimental band at 391 cm^–1^ is not due to an ethene vibration. The calculations predict that this band is a combination of the symmetric and asymmetric Pt–ethene stretches calculated at 360 and 373 cm^–1^. The former is the most intense and, being an *a*-type transition, explains the band’s relative sharpness. It rivals the asymmetric CH_2_ wagging mode at 1027 cm^–1^ in intensity despite having a significantly lower integrated IR intensity. Two bands remain: the weak experimental band at 634 cm^–1^, visible in the inset in Figure 3a, matches a band calculated at 629 cm^–1^, associated with the rocking motion of the whole ethene molecule with respect to Pt. The last experimental band at 1186 cm^–1^ matches a mode possessing both in-phase scissoring and C–C stretch character, here predicted at 1211 cm^–1^ (only Raman-active in free ethene, 1342 cm^–1^). All experimental band frequencies, calculated frequencies, and intensities, including a mode description, are gathered in Table 1.

**Table 1:** Experimental and Calculated Vibrational Frequencies of PtC_2_H_4_^+^ Together with Calculated Intensities and the Assigned Vibrational Mode

frequency (exp, cm^–1^)	frequency (calc, cm^–1^)	intensity (calc, km/mol)	mode description
391	360	14.9	Pt–C stretch (sym)
	373	5.3	Pt–C stretch (asym)
634	629	3.5	twist (sym)
820	803	1.1	rocking (asym)
1030	1027	37.8	wagging (asym)
	1037	4.5	wagging (sym)
1186	1211	5.7	scissoring (sym)
1404	1420	17.0	scissoring (asym)
	1497	2.6	C–C stretch

Upon ethene binding to Pt^+^, the wagging vibrational frequencies are found to increase, whereas a frequency decrease was observed for the C–C stretch and scissoring modes. The lower frequency and thus smaller force constant of the C–C stretch vibration of PtC_2_H_4_^+^ compared to free ethene can be explained by the weakening of the C–C bond by electron donation to Pt^+^. Our calculations show that the C–C bond length increases from 1.325 Å in free ethene to 1.402 Å in PtC_2_H_4_^+^, while the C–H bond length remains virtually the same (1.083 Å in free ethene, 1.085 Å when bound to Pt^+^). Further, upon binding to Pt^+^, the ethene molecule is no longer planar, with all hydrogen atoms lying slightly below the C–C axis and oriented away from the Pt atom. The carbon atoms thus go from sp^2^ hybridization in free ethene to incorporate more sp^3^-like hybridization in PtC_2_H_4_^+^.

The transfer of electron density from ethene to the Pt^+^ ion is also explored by examining the calculated local charges on the atoms as shown in Table 2. A Mulliken population analysis, which is based on electron density present in orbitals of a nucleus, indicates that Pt^+^ has a charge of +0.74e, while ethene has a total charge of +0.26e. This charge on ethene is not equally shared: both carbon atoms sum to −0.52e, and the four hydrogen atoms sum to +0.78e. An alternative metric, the atomic polar tensor (APT) charges, indicates that the net positive charge on Pt in the PtC_2_H_4_^+^ complex equals +0.49e, while the ethene molecule has +0.51e. Here, all carbon and hydrogen atoms have a charge of approximately +0.08e.

**Table 2:** Calculated Mulliken and APT Charges of Single Atoms in PtC_2_H_4_^+^

atoms	Mulliken charges	APT charges
Pt	+0.736	+0.487
C (per atom)	–0.259	+0.079
H (per atom)	+0.196	+0.089

We also observed [Pt,2C,O,6H]^+^ at *m/z* = 240, 241, and irradiation of this ion led to a primary fragment ion at *m/z* = 222, 223 (loss of 18 Da) and a secondary fragment ion at *m/z* = 220, 221 (loss of 20 Da) as identified in Figure S6. As discussed in the Supporting Information, this ion can be identified as (H_2_O)Pt^+^(C_2_H_4_), i.e., the platinum ethene product complexed with adventitious water in the vacuum system. This result further validates the observation of C–C coupling in the present system.

### Reaction Pathway for the Formation of PtC_2_H_4_^+^

**Figure 4:**
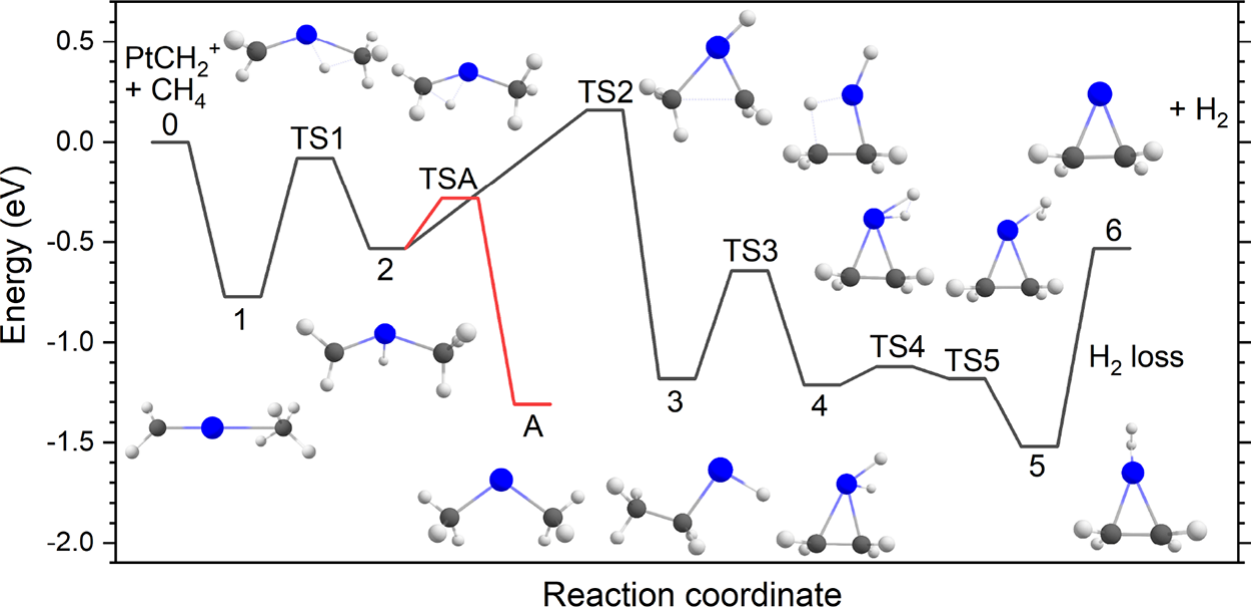
Calculated reaction pathway of the formation of PtC_2_H_4_^+^ from PtCH_2_^+^ and methane using the uB3LYP functional and def2-TZVPPD basis set. The red path shows the formation of Pt(CH_3_)_2_^+^ as observed by Wheeler et al.^24^

To rationalize the formation of ethene when reacting Pt^+^ with two methane molecules, we computationally investigated a potential reaction pathway starting from the platinum carbene product, PtCH_2_^+^, which is formed exothermically by reacting Pt^+^ with one methane molecule as described earlier.^14,15,17^ PtCH_2_^+^ can react with another methane molecule to form the PtC_2_H_4_^+^ product via the pathway indicated by the black trace in Figure 4, with detailed energetics of the intermediates in Table 3. All species shown are on the doublet spin surface, except for methane and dihydrogen, which are singlets. The reaction starts via physisorption of the methane molecule onto PtCH_2_^+^, forming intermediate **1**. The adsorption energy associated with this step is large enough (0.77 eV) to allow transfer of a hydrogen atom from the CH_4_ group to the platinum atom via transition state **TS1**, forming intermediate **2**, HPt(CH_2_)(CH_3_)^+^. Now, the carbon atoms from the carbene and methyl groups can couple together via **TS2**. This step has the highest energy barrier along the path toward PtC_2_H_4_^+^. **TS2** is higher in energy than the PtCH_2_^+^ + CH_4_ reactants by 0.16 eV; however, it still lies 0.21 eV below the energy of the Pt^+^ + 2 CH_4_ reactants. Thus, without dissipation, the energy in the system is sufficient to overcome this barrier. However, if the PtCH_2_^+^ product formed from Pt^+^ and CH_4_ is fully equilibrated with its surroundings, one would expect an equilibrium with at least part of the population trapped before this barrier. Carbon–carbon-coupling via **TS2** leads to intermediate **3**, which is HPt(ethyl)^+^. Transfer of a terminal hydrogen atom from the ethyl group to Pt results in **TS3**, which leads to the formation of a (H)_2_Pt(ethene)^+^ intermediate **4**. Dihydrogen formation on Pt via **TS4** and H_2_ migration in the direction opposite to the ethene ligand via **TS5** results in the formation of a (H_2_)Pt(ethene)^+^ intermediate **5**, which is the lowest-energy species found on this PES. Elimination of the hydrogen molecule at the cost of 0.99 eV results in PtC_2_H_4_^+^, as observed in our experiments.

**Table 3:** Relative Energies (in eV) of the Structures Shown in Figure 4, Taken with Respect to the PtCH_2_^+^ and CH_4_ Reactants

	ion structure	relative energy (eV)
**0**	PtCH_2_^+^ + CH_4_	0
**1**	(CH_4_)PtCH_2_^+^	–0.77
**TS1**		–0.08
**2**	HPt(CH_3_)(CH_2_)^+^	–0.53
**TS2**		+0.16
**3**	HPtCH_2_CH_3_^+^	–1.18
**TS3**		–0.64
**4**	(H)_2_PtC_2_H_4_^+^	–1.21
**TS4**		–1.12
**TS5**		–1.18
**5**	(H_2_)PtC_2_H_4_^+^	–1.52
**6**	PtC_2_H_4_^+^ + H_2_	–0.53
**TSA**		–0.28
**A**	Pt(CH_3_)_2_^+^	–1.31

These results are quite similar to what was obtained by Ye et al., who performed calculations on the activation and dehydrogenation of ethane by Pt^+^ using the less accurate B3LYP/LANL2DZ level of theory.^40^ Our intermediate **3** lies at −1.18 eV (−0.65 with respect to PtC_2_H_4_^+^ + H_2_), a value very close to the −1.16 eV found for a similar structure by Ye et al. The same holds for **TS3** with −0.64 eV (−0.60 eV by Ye et al.) and intermediate **4** with −1.21 eV (−1.19 eV). Intermediate **5**, on the other hand, lies in our investigation at −1.52 eV, while Ye et al. found −1.29 eV. This is also observed for the transition states going from intermediate **4** to **5**, as our energy values lie 0.15 eV lower compared to theirs.^40^

Can this potential energy surface explain why Wheeler et al. found the Pt(CH_3_)_2_^+^ product by reacting Pt^+^ with two methane molecules, while the current study finds PtC_2_H_4_^+^ instead?^24^ The reaction pathway toward Pt(CH_3_)_2_^+^ is shown in red in Figure 4. Starting from intermediate **2**, it is possible to transfer the hydrogen atom on platinum to the carbene group via **TSA**. This leads directly to the formation of the Pt(CH_3_)_2_^+^ species (**A**) found by Wheeler et al. Clearly, the barrier of **TSA** lies well below the PtCH_2_^+^ + CH_4_ reactants and is also lower than the **TS2** barrier. The current calculations match the ones published by Wheeler et al. except for the energetics of the Pt(CH_3_)_2_^+^ species: Wheeler et al. reported a value of −0.92 eV, which we found to be associated to an excited state of ^2^B_1_ symmetry, whereas we found a ^2^A_1_ ground state at −1.31 eV. The ground state has a doubly occupied π* orbital and a singly occupied sd hybrid on Pt, whereas the excited state has a singly occupied π* orbital and a doubly occupied sd hybrid.

From this reaction pathway, we conclude that the higher-pressure environment of the flow-tube reaction cell in the molecular beam experiment used by Wheeler et al. thermalizes the product ions more efficiently. These thermalizing collisions lead to the PtCH_2_^+^ reactant possessing less excess energy such that when it reacts with methane, it cannot easily dehydrogenate. Instead, it gets kinetically trapped at the lowest-energy [Pt,2C,6H]^+^ species, Pt(CH_3_)_2_^+^, that can be formed before **TS2**. In the room-temperature ion trap used here, there are not only fewer thermalizing collisions, but the long storage time and low endothermicity of the +0.16 eV barrier associated with **TS2** also allow the reaction to proceed, and no kinetic trapping of intermediates is observed. The same mild endothermicity may also explain why this reaction was characterized as not facile by Irikura and Beauchamp,^7^ certainly when comparing this barrier to the lack of a barrier for dehydrogenation of the first methane by Pt^+^. The dehydrogenation of CH_4_ by Pt^+^ yields PtCH_2_^+^ + H_2_ and has been experimentally determined to be exothermic by 0.08 ± 0.03 eV.^4^

Finally, we address the fragmentation observed under IR irradiation. Loss of H_2_ from PtC_2_H_4_^+^ to form PtC_2_H_2_^+^ is calculated to require 1.49 eV, not considering any possible barriers, whereas the bond energy between Pt^+^ and C_2_H_4_ is calculated to be 2.94 eV. Thermodynamically, H_2_ loss from PtC_2_H_4_^+^ is thus favored over ethene loss. All values are included in Table 4 together with the energetics of other ions characterized using IRMPD spectroscopy.

**Table 4:** Possible Fragmentation Pathways for the Ions Studied Using IRMPD Spectroscopy

precursor	loss channel	energy (eV)
PtCH_2_^+^	→ PtC^+^ + H_2_	2.50
	→ Pt^+^ + CH_2_ (^3^B_1_)	5.04
	→ Pt^+^ + CH_2_ (^1^A_1_)	5.52
PtC_2_H_4_^+^	→ PtC_2_H_2_^+^ + H_2_	1.49
	→ Pt^+^ + C_2_H_4_	2.94
	→ Pt^+^ + C_2_H_2_ + H_2_	3.24 + 1.49
Pt(C_2_H_4_)_2_^+^	→ Pt(C_2_H_2_)(C_2_H_4_)^+^ + H_2_	1.71
	→ PtC_2_H_4_^+^ + C_2_H_4_	2.11
	→ PtC_2_H_4_^+^ + C_2_H_2_ + H_2_	2.19 + 1.71
	→ PtC_2_H_2_^+^ + C_2_H_4_ + H_2_	1.88 + 1.71
H_2_OPtC_2_H_4_^+^	→ PtC_2_H_4_^+^ + H_2_O	1.73
	→ H_2_OPtC_2_H_4_^+^ + H_2_	1.51
	→ PtC_2_H_2_^+^ + H_2_O + H_2_	1.73 + 1.49
	→ PtH_2_O^+^ + C_2_H_4_	3.05

### IR Spectroscopy of [Pt,4C,8H]^+^

**Figure 5:**
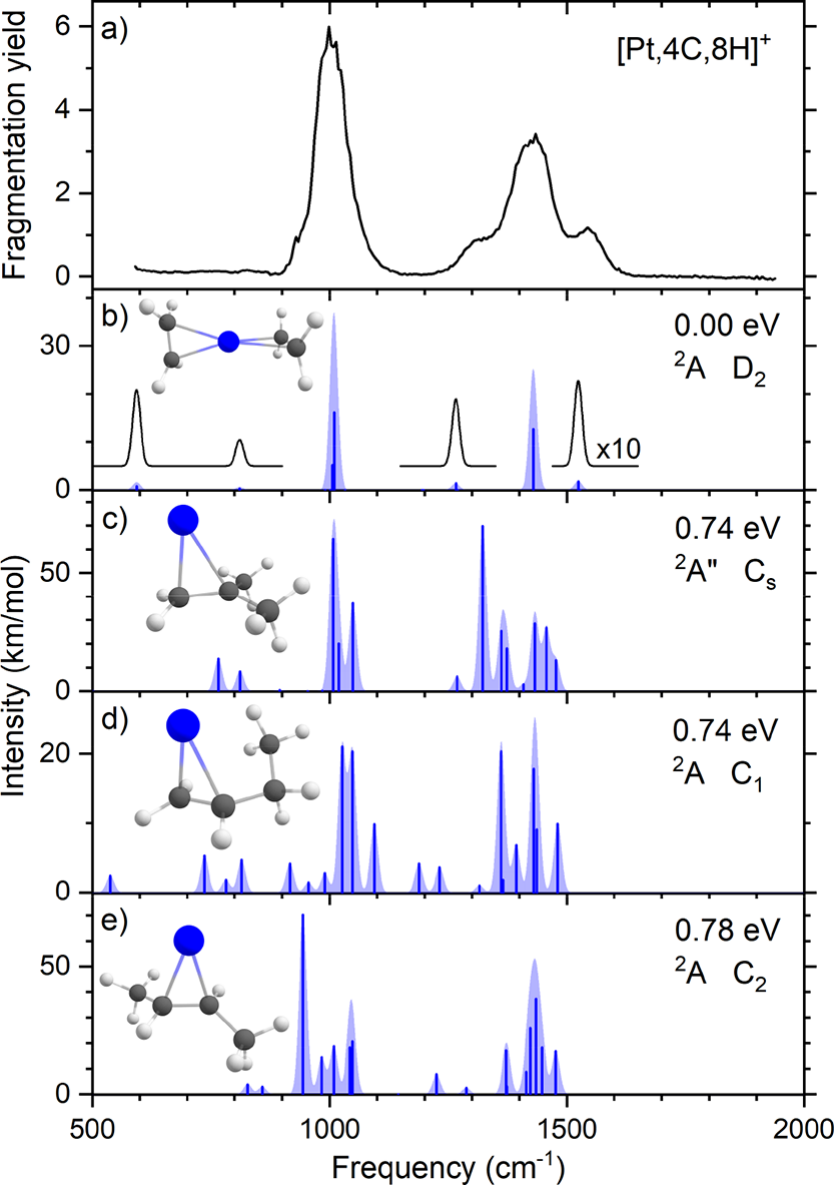
(a) Experimental IRMPD spectrum of [Pt,4C,8H]^+^. (b–e) Calculated spectra of different [Pt,4C,8H]^+^ isomers accompanied by molecular structures, relative energies, electronic ground states, and point groups.

When we increased the methane concentration in the ion trap to 8 × 10^–4^ mbar, we observed a [Pt,4C,8H]^+^ species. In contrast to the case for [Pt,2C,4H]^+^, resonant IR irradiation under moderate intensities led to multiple loss channels: H_2_ loss into the *m/z* = 248, 249 mass channels, C_2_H_4_ loss into the *m/z* = 222, 223 mass channels, and C_2_H_4_ + H_2_ loss into the *m/z* = 220, 221 mass channels. The branching ratio intensities of precursor and fragments as a function of IR frequency are shown in Figure S3. Figure 5a shows the IRMPD spectrum of [Pt,4C,8H]^+^, which contains two strong bands at 998 and 1434 cm^–1^ and two bands of medium intensity at 1315 and 1544 cm^–1^.

The IRMPD spectrum is compared to the calculated spectra of several trial structures. We found a total of 12 stable isomers for the [Pt,4C,8H]^+^ complex, four of which are shown in Figure 5b–e, and the other eight are shown in Figure S4. The calculated isomers shown in Figure 5 were selected based on their relatively low energies and the match of the calculated IR spectrum with the experimental IRMPD spectrum. The structures located have different binding motifs to the Pt^+^ ion as we found σ bonds, π bonds, and long-range interactions for physisorbed methane molecules. Examples of calculated isomers shown in Figure S4 include methane propyne, methane propadiene, methyl allyl, 1- and 2-butene, and hydrido cyclobutyl. All twelve isomers are on the doublet spin surface. The lowest-energy quartet structure found is a PtCH_2_C(CH_3_)_2_^+^ species (isobutene ligand), 2.59 eV higher in energy than the putative global minimum of Pt(C_2_H_4_)_2_^+^.

When comparing the obtained IR spectrum of [Pt,4C,8H]^+^ with the calculated spectra of the isomers selected for Figure 5, we observe a good match with the Pt(C_2_H_4_)_2_^+^ species, the lowest-energy structure. Other species, for instance, the doublet PtCH_2_C(CH_3_)_2_^+^ at 0.74 eV relative to Pt(C_2_H_4_)_2_^+^, exhibit bands at similar frequencies with reasonable intensities, but they do not provide any more satisfactory agreement. The broad structure encompassing the 1315–1434–1544 cm^–1^ triad is tempting to assign as a mixture of species, but we rather suspect that the broad structures are a result of power broadening given the higher pulse energy used here. Rotational envelopes play less of a role for these species as all have rotational constants at most half those of PtC_2_H_4_^+^.

We thus identify the [Pt,4C,8H]^+^ species as a complex containing two ethene ligands that are bound on opposite sides of the Pt, with the C–C bonds oriented at an angle of 90°. The experimental band at 998 cm^–1^ is assigned to be a combination of the symmetric and asymmetric wagging motion of the ethene molecules in Pt(C_2_H_4_)_2_^+^ (see Table 5). The asymmetric wagging motion calculated at 1009 cm^–1^ has a higher intensity compared to the symmetric wagging motion at 1006 cm^–1^. The strong band at 1434 cm^–1^, which is assigned to the asymmetric scissoring vibration, is calculated at a frequency of 1429 cm^–1^. The weaker bands at 1315 and 1544 cm^–1^ are assigned to the C–C stretch and the symmetric scissoring vibrations calculated at 1266 and 1524 cm^–1^.

**Table 5:** Experimental and Calculated Vibrational Frequencies of Pt(C_2_H_4_)_2_^+^ Together with Calculated Intensities and the Assigned Vibrational Mode

frequency (exp, cm^–1^)	frequency (calc, cm^–1^)	intensity (calc, km/mol)	mode description
998	1006	5.2	sym wagging
	1009	32.2	asym wagging
1315	1266	1.4	C–C stretch/scissoring
1434	1429	25.2	asym scissoring
1544	1524	1.8	sym scissoring/C–C stretch

The dominant fragmentation channel of Pt(C_2_H_4_)_2_^+^ is loss of C_2_H_4_ to PtC_2_H_4_^+^. The bond energy of this second ethene molecule is calculated to be 2.11 eV and thus is significantly lower than the bond energy of the first ethene at 2.94 eV. In contrast, the loss of H_2_ resulting in Pt(C_2_H_2_)(C_2_H_4_)^+^ is calculated at 1.71 eV (ignoring the existence of any barriers), making it thermodynamically less favorable than for PtC_2_H_4_^+^ (1.49 eV). The combination of a lower binding energy for the second ethene and a less favorable pathway for H_2_ loss can explain why ethene loss appears to be the dominant loss channel in comparison to H_2_ loss from PtC_2_H_4_^+^. Finally, loss of both H_2_ and a C_2_H_4_ molecule resulting in a PtC_2_H_2_^+^ species requires at least 3.59 eV.

From a catalytic standpoint, the observation that ethene loss is the dominant decomposition pathway for Pt(C_2_H_4_)_2_^+^ is an interesting finding. We can safely assume that H_2_ elimination is not barrierless and that the loss of C_2_H_4_ is the kinetically preferred pathway. This implies that PtC_2_H_4_^+^ itself can be seen as a catalyst for C–H bond activation and C–C coupling in the formation of ethene from two methane molecules. Indeed, the formation of Pt(C_2_H_4_)_2_^+^ + 2 H_2_ from PtC_2_H_4_^+^ + 2 CH_4_ is calculated here to be exothermic by 0.07 eV, although we did not calculate the full potential energy surface. Combined with the 2.11 eV required to remove the second ethene ligand and recover the PtC_2_H_4_^+^ catalyst, this cycle requires a significant energy input of 2.03 eV, but this energy is certainly lower than the original route of cracking followed by Fischer–Tropsch synthesis. It can thus be speculated that similar reaction pathways could be facilitated by supported single-atom catalysts. As such, we expect that the details of the reaction path presented in this work, which appear robust as they can rationalize experimental findings in both low- and high-pressure regimes, can help guide the development of such catalytic systems.

One example of a detail along the reaction path in Figure 4 is the rate-limiting step for C–C coupling, i.e., **TS2**. In the precursor to this transition state, intermediate **2**, the platinum center has three ligands (H, CH_2_, and CH_3_), each of which is bound to the platinum cation by a single covalent bond. This fills the 5d and 6s orbitals on Pt, while the CH_2_ ligand is left with substantial radical character as confirmed by Natural Bond Orbital analysis. The radical character probably facilitates the C–C coupling reaction associated with transfer of methyl from Pt to the CH_2_ radical across **TS2**. This observation may help direct the further exploration of such catalytic chemistry.

## Conclusions

We reacted platinum cations with methane diluted in argon at pressures below 1 × 10^–3^ mbar in an RF ion trap at room temperature. Reactions with different partial pressures of methane led to the formation of platinum ions with different carbon and hydrogen loadings, most prominently [Pt,*n*C,2*n*H]^+^ (*n* = 1, 2, 4) and [Pt,2C,O,6H]^+^. The lack of observation of [Pt,*n*C,(2*n* + 2)H]^+^ products is consistent with earlier low-pressure experiments^5,7,8^ but contrasts with earlier findings where the reaction took place at significantly higher pressures.^24^ IR spectral characterization combined with DFT calculations led to the assignment of [Pt,2C,4H]^+^ as PtC_2_H_4_^+^, demonstrating C–C coupling on atomic platinum cations for the first time. The ethene adduct in this product complex is mildly activated through a weakening of the C=C bond. The reaction path from PtCH_2_^+^ + CH_4_ to PtC_2_H_4_^+^ was calculated, showing a small barrier toward the coupling of the C atoms of +0.16 eV with respect to PtCH_2_^+^ + CH_4_. In the lower-pressure regime of the current experiment, this excess energy is readily available from energy released in the dehydrogenation of the first methane molecule by Pt^+^. It is concluded that the higher-pressure regime in the experiment reported by Wheeler et al. allows a more thorough thermalization of the PtCH_2_^+^ intermediate, preventing the crossing of this barrier. The IR spectral characterization of [Pt,4C,8H]^+^ led to its assignment to Pt(C_2_H_4_)_2_^+^, indicating C–H bond activation of four methane molecules and sequential C–C couplings. IR-induced elimination of an ethene molecule from Pt(C_2_H_4_)_2_^+^ leads to the recovery of the PtC_2_H_4_^+^ species, which can then again react with methane. The lack of observation of a [Pt,3C,6H]^+^ product suggests its facile reactivity with another methane molecule as no pathway without going through this intermediate seems plausible.

## Supporting Information Available

Spectroscopic characterization of the observed [Pt,2C,O,6H]^+^ species; ion intensities, additional isomers and their calculated IR spectra for the [Pt,2C,4H]^+^ and [Pt,4C,8H]^+^ species; calculated energies of formation and vibrational zero-point energies of all presented species and isomers (PDF)
Coordinates, scaled vibrational frequencies, and vibrational intensities for all species and isomers presented (ZIP)

## Notes

The data supporting the findings of this study are deposited in the Radboud Data Repository, https://doi.org/10.34973/t53x-8g50, and is publicly available.
