# Supplementary material for: C–H Bond Activation and C–C Coupling of Methane on a Single Cationic Platinum Center: A Spectroscopic and Theoretical Study
Source: Inorg Chem. 2022 Jul 12;61(29):11252–60. doi: 10.1021/acs.inorgchem.2c01328 (PMC9326971; doi:10.1021/acs.inorgchem.2c01328)
Supplement: Supplementary file 1 — ic2c01328_si_001.pdf [file ic2c01328_si_001.pdf]

C–H bond activation and C–C coupling of methane on a single cationic platinum center: A spectroscopic and theoretical study

Frank J. Wensink[1], Noa Roos[1], Joost M. Bakker[1,*], P. B. Armentrout[2,*]

[1]Radboud University, Institute for Molecules and Materials, FELIX Laboratory, Toernooiveld 7, 6525ED Nijmegen, The Netherlands
[2]University of Utah, Department of Chemistry, 315 South 1400 East, Room 2020, Salt Lake City, Utah 84112, United States

*Corresponding authors: joost.bakker@ru.nl, armentrout@chem.utah.edu

SUPPORTING INFORMATION

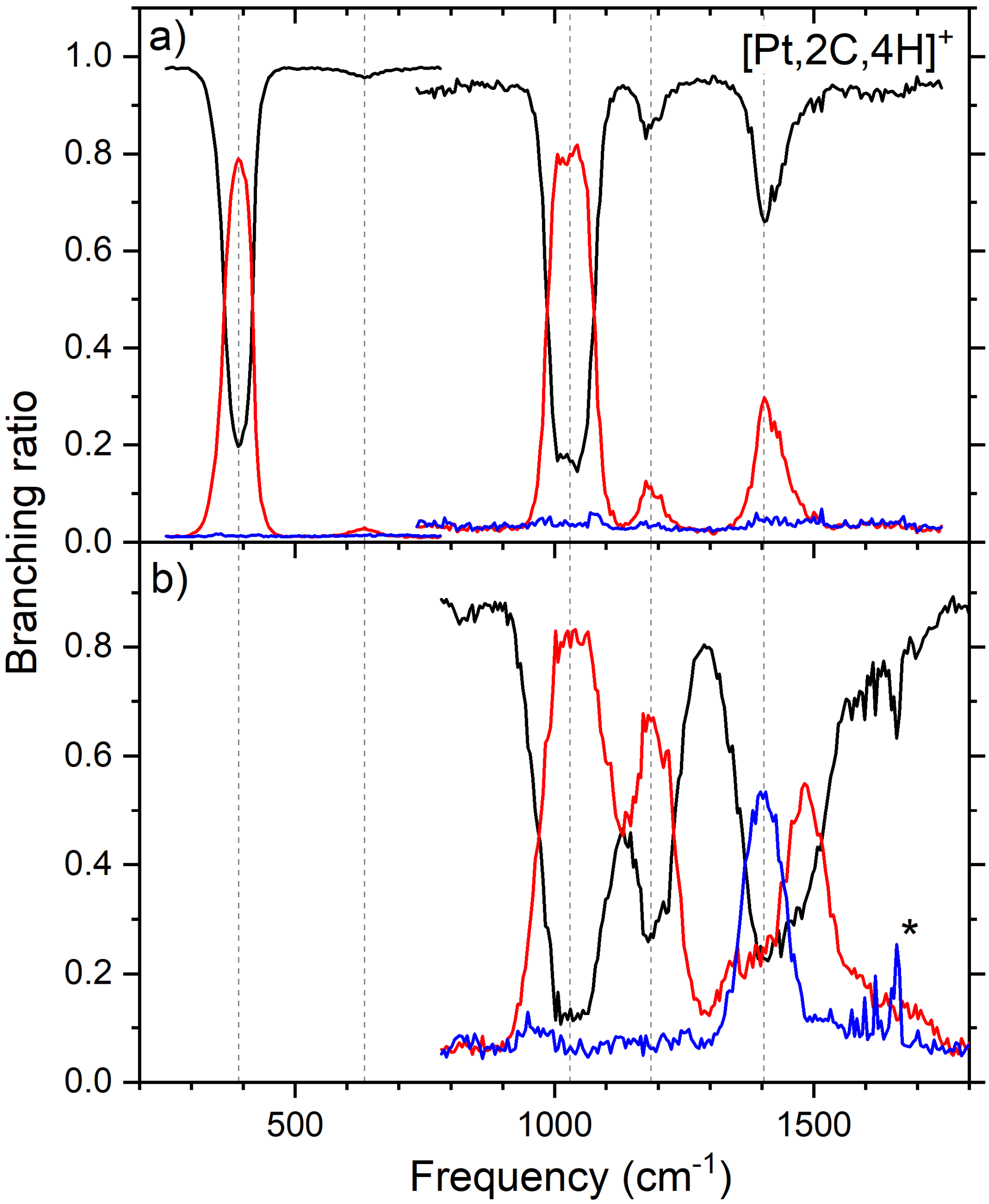

Figure S1: Branching ratios of the precursor $PtC_2H_4^+$ (black traces, *m/z* = 222, 223) and fragment ions $PtC_2H_2^+$ (red, *m/z* = 220, 221), and Pt^+ (blue, *m/z* = 194,195) as a function of IR frequency at reduced (panel a) and higher (panel b) IR intensities. Dashed vertical lines indicate the absorption maxima for the precursor species. Artifacts due to electrical noise pickup obscuring the mass of interest are denoted by an asterisk (*).

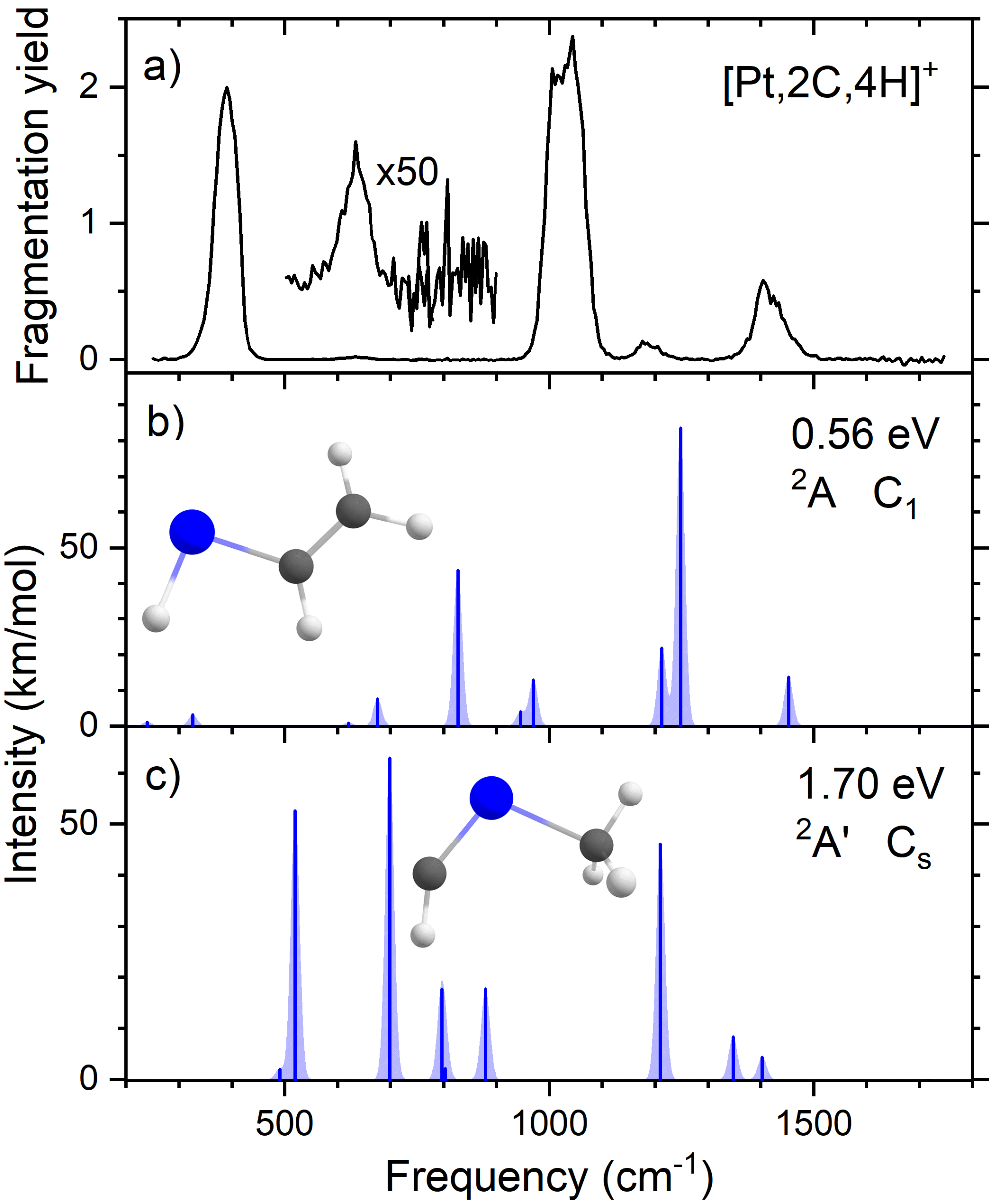

Figure S2: a) Experimental IRMPD spectrum of $[Pt,2C,4H]^+$, recorded between 250 and 780 cm^{-1} in ICR cell 3 and between 735 and 1745 cm^{-1} in ICR cell 4; b) and c) Calculated spectra of different $[Pt,2C,4H]^+$ isomers accompanied by molecular structures, relative energies, electronic ground states and point groups.

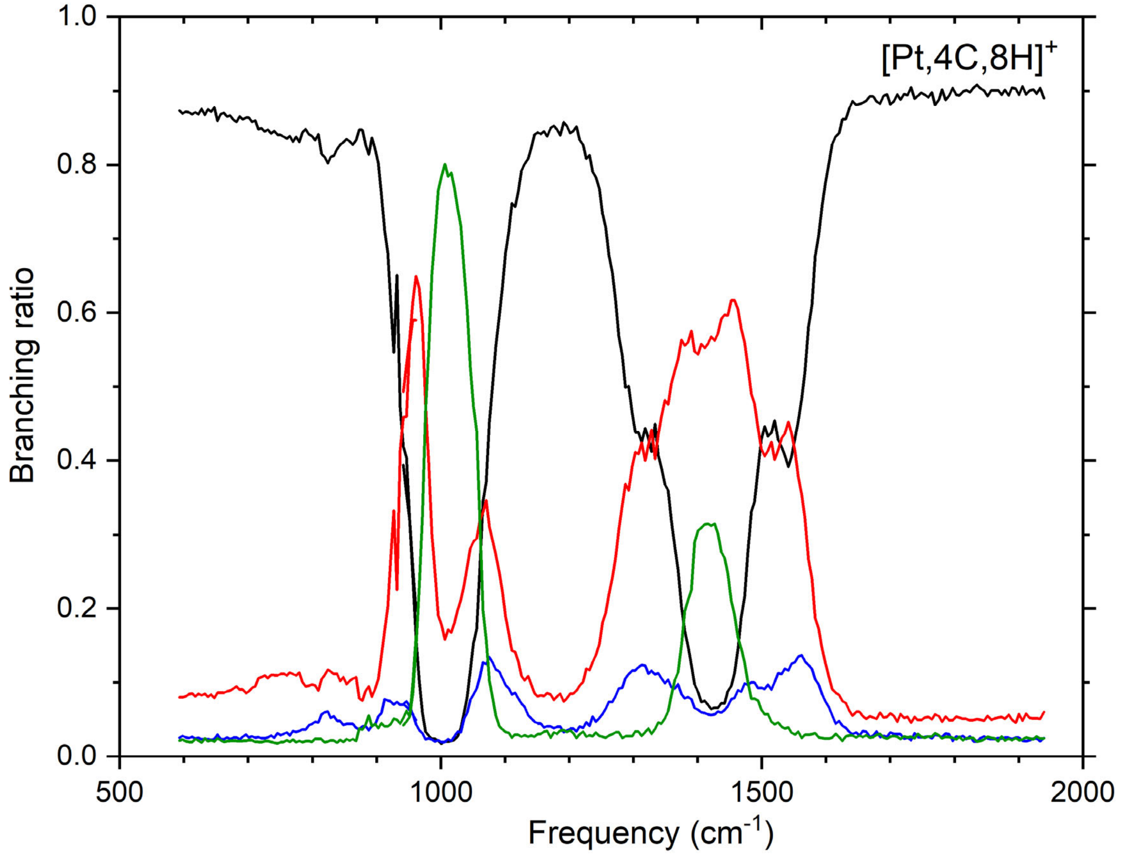

Figure S3: Branching ratios of the precursor $Pt(C_2H_4)_2^+$ (black trace, *m/z* = 250, 251) and fragment ions $PtC_2H_4^+$ (red trace, *m/z* = 222, 223), $Pt(C_2H_2)(C_2H_4)^+$ (blue trace, *m/z* = 248, 249), and $PtC_2H_2^+$ (green trace, *m/z* = 220, 221) as a function of IR frequency.

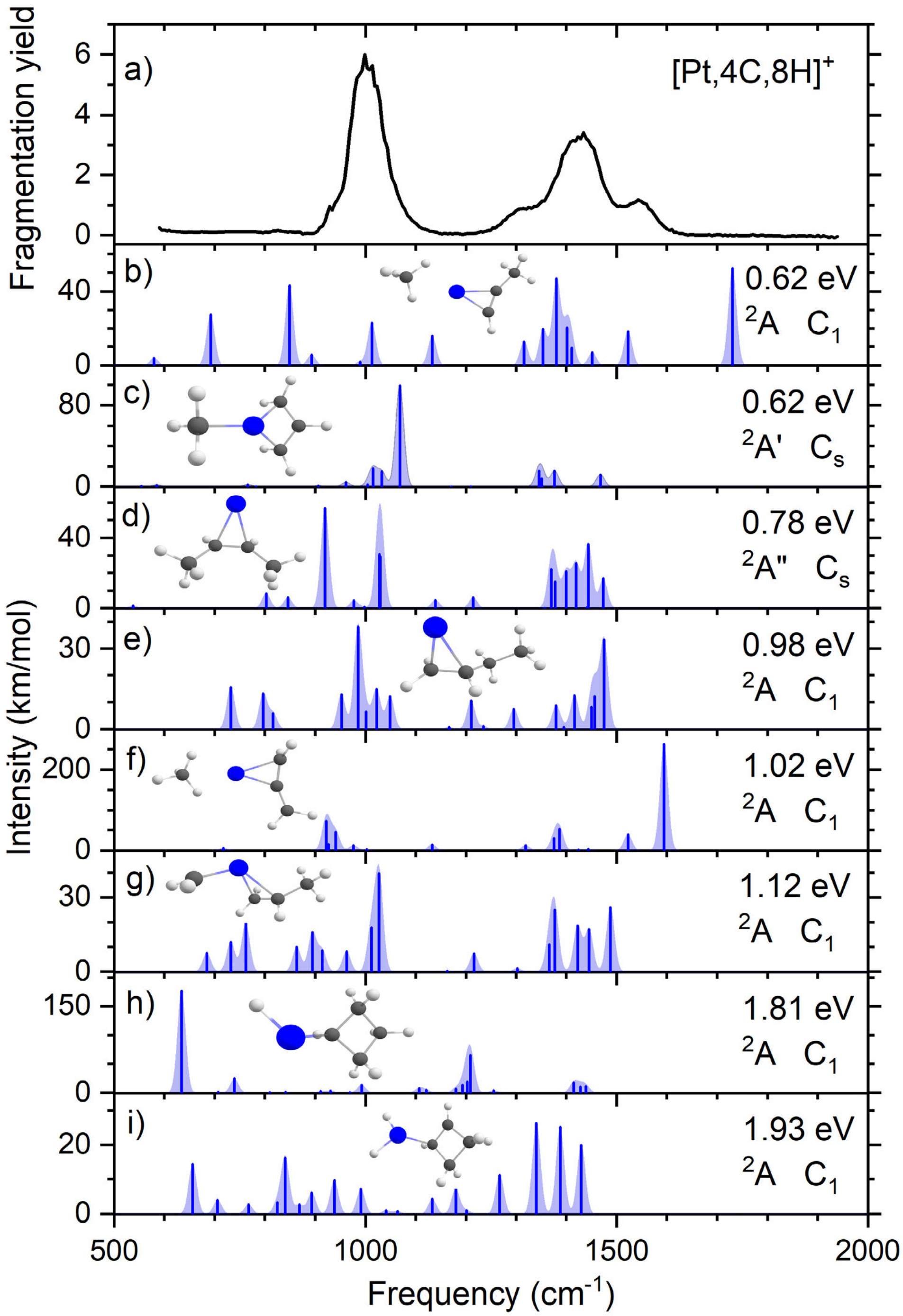

Figure S4: a) Experimental IRMPD spectrum of $[Pt,4C,8H]^+$. b-i) Calculated spectra of $[Pt,4C,8H]^+$ isomers not shown earlier accompanied by molecular structures, relative energies, electronic ground states and point groups.

IR spectroscopy of $[Pt,2C,O,6H]^+$

The IRMPD spectrum of $[Pt,2C,O,6H]^+$ is shown in Figure S5a. Irradiation of $[Pt,2C,O,6H]^+$ at *m/z* = 240, 241 led to a primary fragment ion at *m/z* = 222, 223 (loss of 18 Da) and a secondary fragment ion at *m/z* = 220, 221 (loss of 20 Da), as identified in Figure S6. Intense bands were found at 1022, 1415, and 1622 cm^{-1} along with weaker bands at 1252 and 1528 cm^{-1}.

Calculated IR spectra of several isomers of $[Pt,2C,O,6H]^+$ are shown in Figure S5b-g. The most stable isomer found is $H_2OPtC_2H_4^+$, which has a $^2A'$ ground state, with a $^2A''$ excited state only 0.22 eV higher in energy. Other isomers that are higher in energy are dihydride acetaldehyde, $PtC_2H_4^+$ with water bound to the ethene ligand, an ethanol ligand, and a dimethyl ether ligand. All relevant isomers are found on the doublet spin surface. The lowest-energy quartet spin state found is 2.95 eV above the putative global minimum and has a $H_2OPtC_2H_4^+$ structure, even though the water and ethene ligands have a different bonding pattern to Pt compared to the doublet state. When we compare the calculated IR spectra with the experimentally obtained IRMPD spectrum, the best match is observed for the $H_2OPtC_2H_4^+$ ground state structure. All bands observed, including the two minor ones, can be assigned to this structure. The experimental band at 1022 cm^{-1} matches the ethene wagging modes calculated at 1026 cm^{-1}, and the experimental band at 1252 cm^{-1} matches the C–C stretch calculated at 1233 cm^{-1}. The experimental bands at 1415 and 1528 cm^{-1} are assigned to the asymmetric and symmetric ethene scissoring motions, respectively. The experimental band at 1622 cm^{-1} is assigned to the water bending mode calculated at 1595 cm^{-1} (also 1595 cm^{-1} for free water[1]). All experimental and calculated frequencies are included in Table S1.

The fragmentation behavior of $H_2OPtC_2H_4^+$ shown in Figure S6 can now be understood. Loss of 18 Da after resonant IR irradiation corresponds to the loss of water. The bond energy between water and $PtC_2H_4^+$ is calculated to be 1.73 eV, while the bond energy between ethene and PtH_2O^+ is 3.05 eV. This explains the preferred loss of water over loss of ethene. The loss channel of 20 Da then corresponds to a higher energy channel of sequential H_2O and H_2 loss.

(1) Shimanouchi, T. Tables of Molecular Vibrational Frequencies. Consolidated Volume I. *National Bureau of Standards*. National Bureau of Standards 1972, pp 1–160.

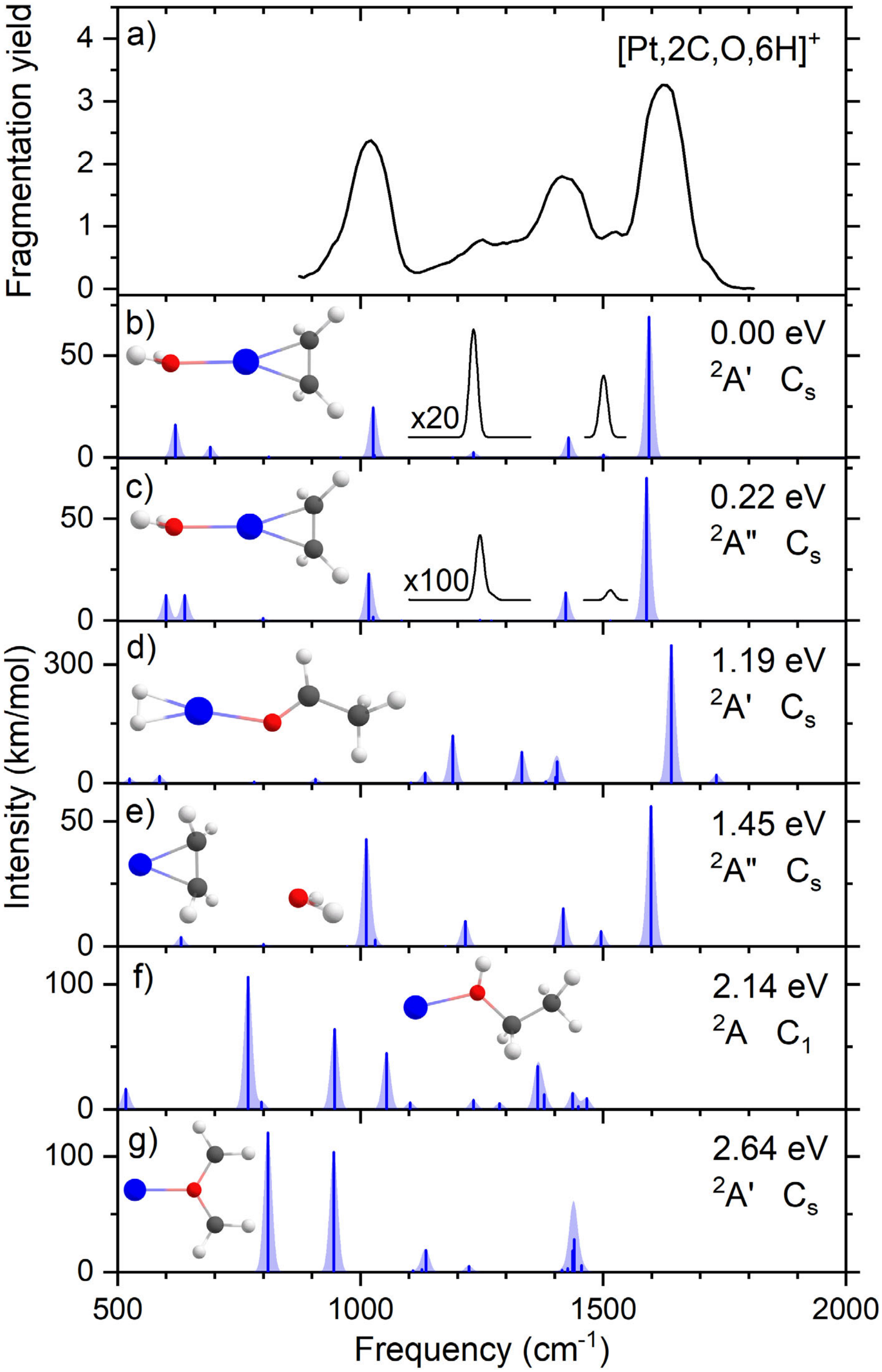

Figure S5: a) Experimental IRMPD spectrum of $[Pt,2C,O,6H]^{+}$; b-g) Calculated spectra of different $[Pt,2C,O,6H]^{+}$ isomers accompanied by molecular structures, relative energies, electronic ground states and point groups.

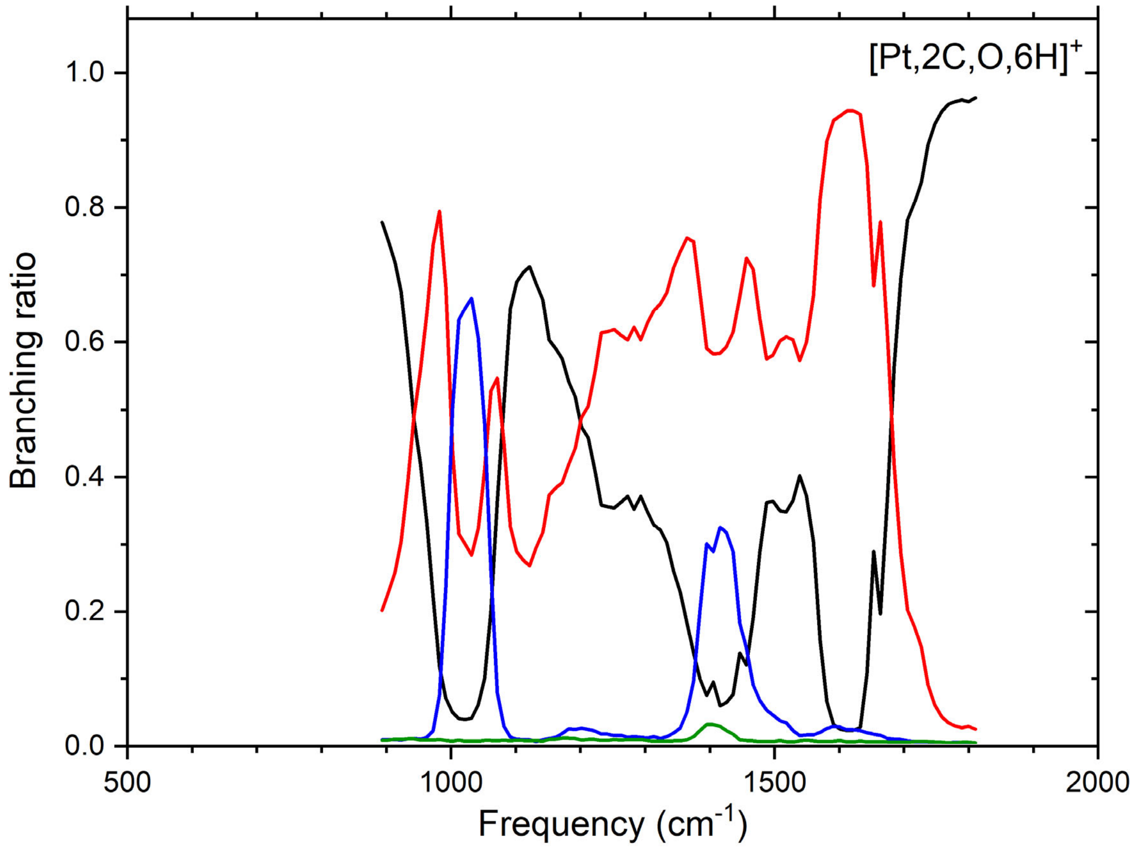

Figure S6: Branching ratios of the precursor $H_2OPtC_2H_4^+$ (black trace, *m/z* = 240, 241) and fragment ions $PtC_2H_4^+$ (red trace, *m/z* = 222, 223), $PtC_2H_2^+$ (blue trace, *m/z* = 220, 220), and Pt^+ (green trace, *m/z* = 194, 195) as a function of IR frequency.

Frequency (exp, cm^{-1})	Frequency (calc, cm^{-1})	Intensity (calc, km/mol)	Mode description
1022	1026	24.5	Ethene wagging asym (& sym)
1252	1233	2.7	C-C stretch
1415	1429	9.9	Ethene scissoring asym
1528	1501	1.5	Ethene scissoring sym
1622	1595	69.2	Water scissoring

Table S1: Experimental and calculated vibrational frequencies of $H_2OPtC_2H_4^+$ together with calculated intensities and the assigned vibrational mode.

Geometry	E (Hartree)	ZPE	ΔE (eV)
Fig 3b: $PtC_2H_4^+$	-197.7081796	0.0535158	0
Fig 3c: $PtCHCH_3^+$	-197.6968308	0.0511285	0.24
Fig 3d: cis-$HPtCHCH_2^+$	-197.6817034	0.047291	0.55
Fig 3e: $(H_2)PtC_2H_2^+$	-197.6678611	0.0431414	0.81
Fig 3f: $Pt(CH_2)_2^+$	-197.6459823	0.0469108	1.51
Fig S2b: trans-$HPtCHCH_2^+$	-197.6814411	0.0473796	0.56
Fig S2c: $HCPtCH_3^+$	-197.6400164	0.0478276	1.70

Table S2: Energetics of $[Pt,2C,4H]^+$ species. The XYZ coordinates, scaled vibrational frequencies and vibrational intensities can be found in Supporting Information 2 (ZIP).

Geometry	E (Hartree)	ZPE	ΔE (eV)
Fig 5b: $Pt(C_2H_4)_2^+$	-276.4144175	0.1077687	0
Fig 5c: Pt(isobutylene)$^+$	-276.3886468	0.1090855	0.74
Fig 5d: Pt(1-butene)$^+$	-276.3885096	0.1090403	0.74
Fig 5e: Pt(2-butene)$^+$	-276.3875337	0.1094338	0.78
Fig S4b: (CH_4)Pt(propyne)$^+$	-276.3861021	0.1021421	0.62
Fig S4c: H_3CPt(allyl)$^+$	-276.3886392	0.1046943	0.62
Fig S4d: Pt(2-butene)$^+$	-276.3872135	0.1093945	0.78
Fig S4e: Pt(1-butene)$^+$	-276.3804502	0.1098093	0.98
Fig S4f: (CH_4)Pt(propadiene)$^+$	-276.3710827	0.1019512	1.02
Fig S4g: H_2CPt(propene)$^+$	-276.3705097	0.105092	1.12
Fig S4h: HPt(cyclobutyl)$^+$	-276.3456110	0.1055298	1.81
Fig S4i: HHPt(cyclobutene)$^+$	-276.3362086	0.1004823	1.93

Table S3: Energetics of $[Pt,4C,8H]^+$ species. The XYZ coordinates, scaled vibrational frequencies and vibrational intensities can be found in Supporting Information 2 (ZIP).

Geometry	E (Hartree)	ZPE	ΔE (eV)
Fig S5b: $(H_2O)PtC_2H_4^+$	-274.2452702	0.0788268	0
Fig S5c: $(H_2O)PtC_2H_4^+$	-274.2376036	0.0791585	0.22
Fig S5d: (H_2)Pt(acetaldehyde)$^+$	-274.1962657	0.0734173	1.19
Fig S5e: $PtC_2H_4(H_2O)^+$	-274.1896722	0.0765566	1.45
Fig S5f: Pt(ethanol)$^+$	-274.1687380	0.0810644	2.14
Fig S5g: Pt(dimethyl ether)$^+$	-274.1506605	0.0811597	2.64

Table S4: Energetics of $[Pt,2C,O,6H]^+$ species. The XYZ coordinates, scaled vibrational frequencies and vibrational intensities can be found in Supporting Information 2 (ZIP).

Geometry	E (Hartree)	ZPE	ΔE (eV)	TS freq (cm^{-1})
**0**: CH_4	-40.5391556	0.0445911	x	
**0**: $PtCH_2^+$	-158.3335976	0.0231867	0	
**1**: $(CH_4)PtCH_2^+$	-198.9027168	0.0695908	-0.77	
**TS1**: $CH_3HPtCH_2^+$	-198.8738668	0.065825	-0.08	-837
**2**: $CH_3PtHCH_2^+$	-198.8910948	0.0666062	-0.53	
**TSA**: $CH_3PtHCH_2^+$	-198.8804621	0.0652112	-0.28	-825
**A**: $Pt(CH_3)_2^+$	-198.9246675	0.0714715	-1.31	
**TS2**: $HPtCH_2CH_3^+$	-198.8661002	0.0670031	+0.16	-363
**3**: $HPtCH_2CH_3^+$	-198.9183714	0.0701142	-1.18	
**TS3**: $HHPtC_2H_4^+$	-198.8938878	0.0655442	-0.64	-968
**4**: $HHPtC_2H_4^+$	-198.9161919	0.0668282	-1.21	
**TS4**: $HHPtC_2H_4^+$	-198.9110157	0.06498	-1.12	-807
**TS5**: $(H_2)PtC_2H_4^+$	-198.9150540	0.0666436	-1.18	-251
**5**: $(H_2)PtC_2H_4^+$	-198.9303123	0.0693388	-1.52	
**6**: H_2	-1.1800213	0.0100724	x	
**6**: $PtC_2H_4^+$	-197.7081796	0.0535158	-0.53	

Table S5: Energetics of species present on the potential energy surface of Figure 4. Transition state frequencies shown here are unscaled. The XYZ coordinates, scaled vibrational frequencies and vibrational intensities can be found in Supporting Information 2 (ZIP).
